# Seq-ing improved gene expression estimates from microarrays using machine learning

**DOI:** 10.1186/s12859-015-0712-z

**Published:** 2015-09-04

**Authors:** Paul K. Korir, Paul Geeleher, Cathal Seoighe

**Affiliations:** 1 School of Biochemistry and Cell Biology, University College Cork, Western Road, Cork, Ireland; 2 Section of Hematology/Oncology, Department of Medicine, University of Chicago, Chicago, IL-60637 USA; 3 School of Mathematics, Statistics and Applied Mathematics, University Road, Galway, Ireland; 4 Institute of Infectious Disease and Molecular Medicine, Anzio Road, Cape Town, 7925 South Africa

**Keywords:** RNA-Seq, Microarray, Machine learning, Statistical learning

## Abstract

**Background:**

Quantifying gene expression by RNA-Seq has several advantages over microarrays, including greater dynamic range and gene expression estimates on an absolute, rather than a relative scale. Nevertheless, microarrays remain in widespread use, demonstrated by the ever-growing numbers of samples deposited in public repositories.

**Results:**

We propose a novel approach to microarray analysis that attains many of the advantages of RNA-Seq. This method, called *Machine Learning of Transcript Expression (MaLTE)*, leverages samples for which both microarray and RNA-Seq data are available, using a Random Forest to learn the relationship between the fluorescence intensity of sets of microarray probes and RNA-Seq transcript expression estimates. We trained MaLTE on data from the Genotype-Tissue Expression (GTEx) project, consisting of Affymetrix gene arrays and RNA-Seq from over 700 samples across a broad range of human tissues.

**Conclusion:**

This approach can be used to accurately estimate absolute expression levels from microarray data, at both gene and transcript level, which has not previously been possible. This methodology will facilitate re-analysis of archived microarray data and broaden the utility of the vast quantities of data still being generated.

**Electronic supplementary material:**

The online version of this article (doi:10.1186/s12859-015-0712-z) contains supplementary material, which is available to authorized users.

## Background

Much effort has been invested in developing accurate methods to infer gene expression levels from the fluorescence intensities of microarray probes. Typical analysis pipelines for oligonucleotide microarrays include subtraction of background signals, normalization of the signal intensities across samples and summarization of the fluorescence intensities of probes that map to the same genomic feature (gene or transcript) [1, 2]. Numerous approaches have been applied to obtain gene-level expression estimates from probe intensities [3] with robust linear models (RMA, parameter estimation by median-polish) and multiplicative models (PLIER, MAS5.0, and dChip) suggested to give the best results [4]. Methods that directly leverage existing datasets have also been proposed, for example, sequencing scaled microarray intensities (SSMI) employs quantile-based scaling to produce intensities that result in higher statistical power to infer differential expression [5].

Although they remain in very widespread use, microarrays have a number of limitations in comparison to sequencing-based methods for the quantification of gene expression (generally referred to as RNA-Seq). Documented advantages of RNA-Seq include improved reproducibility and dynamic range and gene expression estimates on an absolute scale [6]. The latter attribute facilitates comparison of gene expression levels between different experiments and also enables the expression of different genes within the same sample to be compared, which is useful, for instance in modeling regulatory networks [6]. Currently, the summarized probe intensity values obtained from microarrays, by contrast, are on an arbitrary scale, cannot easily be compared between experiments without renormalization and are not well suited to the comparison of expression levels of different genes in the same sample [7–9]. In addition, the chips are designed according to specific genome annotations and need to be adapted when these annotations change. Finally, the dynamic range of microarrays (a few hundred fold) is an order of magnitude lower than that of RNA-Seq (approx. 9,000 fold depending on the coverage) making them less sensitive [10]. However, because of their relatively low cost, microarrays remain in widespread use and are particularly important for large-scale studies and in smaller and less well-funded laboratories [11–13].

Here we investigate an alternative approach to estimating gene or transcript expression levels from microarray probe level fluorescence intensities, which addresses many of the limitations discussed above. We refer to this method as machine learning of transcript expression (MaLTE). Given a set of samples for which both microarray and RNA-Seq data are available, MaLTE uses machine-learning techniques to learn the relationship between the intensities of a set of probes associated with the gene and the expression level of the feature as estimated by RNA-Seq. The regression models can then be applied to estimate gene expression from microarray data for which RNA-Seq data is not available. We applied MaLTE to 716 samples from a broad range of tissues profiled by the Genotype-Tissue Expression (GTEx) project, training the regression models on a random subset of the samples and testing on the remainder. Within individual test samples the gene expression estimates from MaLTE approximate closely the values estimated by RNA-Seq. Cross-sample correlation between RNA-Seq and microarray expression estimates for individual genes was also significantly higher using MaLTE compared to existing methods to estimate gene expression from microarrays. Remarkably, MaLTE can also leverage transcript isoform expression estimates produced from RNA-Seq to provide a means to estimate the expression levels of specific isoforms from the microarray data.

## Materials and Methods

### GTEx data preparation

Affymetrix Human Gene 1.1 ST microarray and RNA-Seq expression data (derived from 837 samples across 29 tissue types and three cell lines) were downloaded from the genotype-tissue expression (GTEx) project website (http://www.broadinstitute.org/gtex) on 30^th^ July 2013. For 91 of the samples, only microarray data was available and multiple biological replicates were included for several of the cell lines. A total of 716 of the samples were used in this study.

Raw and RMA (median-polish) processed microarray data were downloaded from GEO (accession GSE45878). Library files for Affymetrix Human Gene 1.1 ST arrays were downloaded from the Affymetrix website (http://www.affymetrix.com). Custom CDF files for Human Gene (HuGene11stv1_Hs_ENSG.cdf) that were used in the GTEx project [14] were also downloaded from the Brainarray resource (http://brainarray.mbni.med.umich.edu). Probes were extracted using Affymetrix Power Tools (APT v1.12.0) with and without quantile-normalization and with background correction (http://www.affymetrix.com/estore/partners_programs/programs/developer/tools/powertools.affx). Expression estimates obtained using RMA were downloaded from GEO. Gene expression was also estimated using PLIER [15] as implemented in APT using the custom CDF (HuGene11stv1_Hs_ENSG.cdf).

We mapped genes to probes using two datasets: (i) the mapping between probes and probe sets implied within the probe intensities file and (ii) the mapping between probe sets and genes. The data for (ii) were constructed using the BEDTools intersect utility. This utility takes two BED files as input: one for gene and another for probe set genomic coordinates. Gene coordinates were determined from the Ensembl v.72 gene model [16] while probe set coordinates were downloaded from the Affymetrix website (http://www.affymetrix.com).

Altogether, we assessed expression estimates for 26,215 genes. These corresponded to 140,212 transcript isoforms. Quantile normalization of gene RPKM values for the 26,215 genes was performed prior to training and testing. MaLTE as well as RMA and PLIER expression estimates were also quantile-normalized prior to downstream analyses.

### Selecting and tuning the best learning algorithm

We used an independent dataset to select and tune the optimal learning algorithm. We obtained RNA-Seq data [17, 18] and Affymetrix Human Exon 1.0 ST array CEL files [19] for 53 Yoruba (YRI) and 44 European (CEU) HapMap cell lines [20]. All individuals were unrelated. Data was downloaded from the GEO database [21] under accessions GSE25030 (CEU RNA-Seq), GSE19480 (YRI RNA-Seq) and GSE7851 (CEU and YRI exon array data).

We compared the performance of several learning algorithms: *classification and regression trees (CART)*, *multivariate adaptive regression splines (MARS)*, *boosted regression trees (BRT)*, *random forest (RF)*, *conditional random forest (CRF)* and *quantile regression random forest (QRF)* [22–26]. All learning algorithms were compared to median-polish and PLIER. All methods were applied in the R statistical computing environment [27]. Performance was evaluated based on the mean cross-sample correlation over all genes (see *Results* for a definition).

We identified CRF as the best performing algorithm and then optimized its performance by identifying the best parameter settings. To do this, we created ten datasets, each with 1,000 randomly selected genes. We examined the following parameters: *minimum number of features (probes) selected* (*FS*), *number of randomly sampled predictor probes* (mtry) and *number of trees* (ntree). We chose the optimal value of each parameter based on the mean cross-sample Pearson correlation coefficient of out-of-bag (OOB) estimates for positively correlated genes (*r*_*OOB*_>0). We identified the optimal *FS* by restricting to the *n* probes that were most highly correlated with the response in the training set. We also identified quantile regression random forest (QRF) as having equivalent performance with the added benefit of producing prediction intervals (upper and lower quantiles). Tuned parameters for the best algorithm were applied without modification on the GTEx dataset.

### Application to archived samples

A dataset consisting of Affymetrix Human Exon 1.0 ST array and RNA-Seq expression estimates from a set of human brain samples was downloaded from GEO (accession GSE26586) [28]. To apply MaLTE, trained on Affymetrix Human Gene 1.1 ST arrays, to data from the Affymetrix Human Exon 1.0 ST arrays, we needed to map probes shared between the two platforms using comparison spreadsheets. Spreadsheets relating exon array to gene array meta-probe sets were downloaded from the Affymetrix website (http://www.affymetrix.com). We used meta-probe sets classified as “Best Match” to map exon array probes to gene array probes. A probe was converted if it was part of a probe set that was in a meta-probe set in the “Best Match” spreadsheet and only if it shared 100% sequence similarity between arrays. In total 425,268 of the 861,493 probes on the gene array could be mapped to exon array probes in this way. GTEx and brain RNA-Seq and probe intensities were then quantile-normalized together but we excluded background correction because the process of transforming the exon arrays excluded several background probes, which would hamper comparison to non-background-corrected median-polish and PLIER.

To compare performance between median-polish, PLIER and MaLTE, we redefined the exon array meta-probe set mappings. (For a detailed description of the exon array design please refer to [29]). We did this to ensure that the same gene-to-probe set definitions were applied to all methods. Meta-probe sets represent the gene/transcript level and are quantified by summarizing the estimates from the individual probe sets. For each Ensembl gene identifier, we identified all exonic probe sets using xmapcore (now annmap) [30]. An exonic probe set is defined as having: (i) all probes mapping to the genome only once, and (ii) all probes falling within an exon boundary. We then used the Ensembl gene identifiers as meta-probe set identifiers. We also constructed a text file of ‘kill list’ (APT option: –kill-list) probes, which consisted of probes that were excluded from the modified exon array. Gene expression estimates were then computed using APT for custom and core meta-probe sets.

### Application to the Affymetrix tissue mixture dataset

The Affymetrix tissue mixture dataset was downloaded from the Affymetrix website (http://www.affymetrix.com). This consisted of nine mixtures of brain and heart in varying proportions (1:0 (heart to brain), 0.95:0.5, 0.9:0.1, 0.75:0.25, 0.5:0.5 (three biological replicates), 0.25:0.75, 0.1:0.9, 0.05:0.95, and 0:1) with three technical replicates of each. Because this dataset consisted of Affymetrix Human Gene 1.0 ST arrays, we mapped probes to Human Gene 1.1 ST probe identifiers. The same custom library files were used to evaluate median-polish and PLIER summarization after background correction and quantile normalization. Tissue mixture proportions were then estimated using the R package CellMix [31] for common genes following OOB filtering.

## Results

We hypothesized that learning the relationship between single-channel microarray probe intensities and RNA-Seq expression estimates from samples for which microarray and RNA-Seq data are available could provide a means to obtain improved estimates of gene expression from microarrays. We refer to this general approach as MaLTE. To investigate the performance of the MaLTE approach we applied it to 716 samples from a broad range of human tissues for which high quality Affymetrix Human Gene 1.1 ST microarray and RNA-Seq data have been generated through the pilot phase of the GTEx project [14]. We selected approximately one-fifth (146) of the 716 unique samples at random for use as the training set and tested the performance of MaLTE on the remaining samples. Training on a larger number of samples did not lead to substantial improvement in prediction accuracy (Supplementary Fig. S1). For each feature of interest (gene or transcript), we identified a set of probes with genomic co-ordinates that overlap the coordinates of the feature and used quantile regression random forest, including a feature selection step, to learn the relationship between the RNA-Seq expression estimates and the probe intensities in the training data (see *Online Methods* for details of the choice of regression algorithm). MaLTE is available as an R software package from http://bioinf.nuigalway.ie/MaLTE/malte.html.

### Correlation with RNA-Seq

**Fig. 1:**
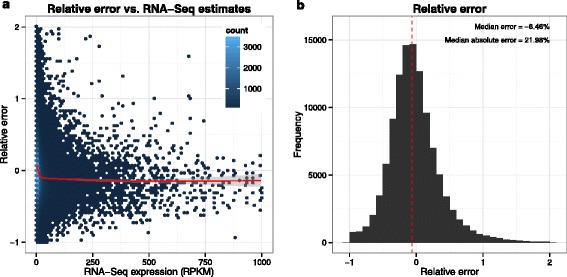
Estimation accuracy. MaLTE provides an estimate of the RNA-Seq gene expression levels from microarray probe intensities. (**a**) The relative error (i.e. difference between MaLTE estimate and RNA-Seq, divided by the RNA-Seq value) as a function of the RNA-Seq expression level. Each point corresponds to a bin of 75 genes. The data represents all genes but with a random subset of 10 samples for each gene. Only relative errors below 2 and RNA-Seq values between 1 and 1000 are represented. Low expression genes were excluded due to high stochasticity for low read counts. A Loess regression line is shown in red, illustrating that MaLTE slightly underestimates RNA-Seq particularly for highly-expressed genes. (**b**) The distribution of relative error with percentage median error and median absolute error displayed with the median error indicated by the dashed red line

**Fig. 2:**
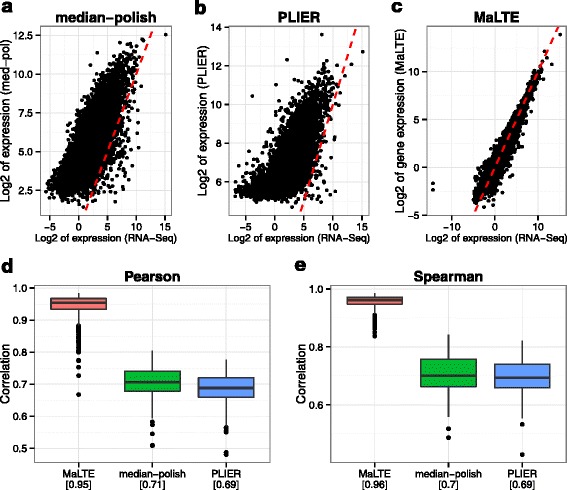
Within-sample correlation with RNA-Seq. (**a**, **b**, **c**) Scatter plot of gene expression for a single exemplary sample for each method against RNA-Seq. The sample with the within-sample Pearson correlation closest to the median over all samples was chosen. Box plots are provided to show the range of within-sample (**d**) Pearson and (**e**) Spearman correlation coefficients across samples in the test dataset. Median correlations are indicated beneath in brackets

Given a putatively accurate measure of gene expression (here RNA-Seq), our goal is to approximate this measure from the array probe intensities. For most genes we could estimate the RNA-Seq expression level, given as reads per kilobase of transcript per million mapped reads (RPKM) relatively accurately (Fig. 1). For the lowest expressed genes, accuracy is limited by stochastic fluctuation in the number of reads from a given transcript and for the highest expressed genes we underestimate expression level due to saturation of microarray probe intensities. We also calculated the correlation between MaLTE and RNA-Seq and compared it to the correlations with RNA-Seq obtained from two existing widely-used methods to estimate expression from sets of microarray oligonucleotide probe intensities (median polish [2] and PLIER [15]). Comparison was carried out both for correlation within samples and across samples. The former provides an indication of the agreement between the methods on the relative expression of different genes within the same sample, while the latter is an indication of how well the variation across samples detected by RNA-Seq is captured by the array estimates. Strikingly, gene expression levels estimated by MaLTE on the test samples showed dramatically higher within-sample Pearson correlation with the RNA-Seq estimates than median-polish or PLIER (Fig. 2a–2d). Importantly, the improved within-sample Pearson correlation is not simply a result of MaLTE rescaling the microarray expression estimates to match the RNA-Seq data, as MaLTE also results in substantially higher within-sample rank correlation (Fig. 2e). The slopes of the within-sample regression lines were close to unity for MaLTE (e.g. Fig. 2c). In contrast, the expression estimates from the other methods are not on the same scale as the RNA-Seq values (Fig. 2a and 2b). By placing gene expression estimated from the arrays on the absolute scale defined by RNA-Seq, MaLTE allows comparison of gene expression between genes on the array. This is not possible with standard summarization techniques, such as median-polish and PLIER [6, 8, 9].

**Fig. 3:**
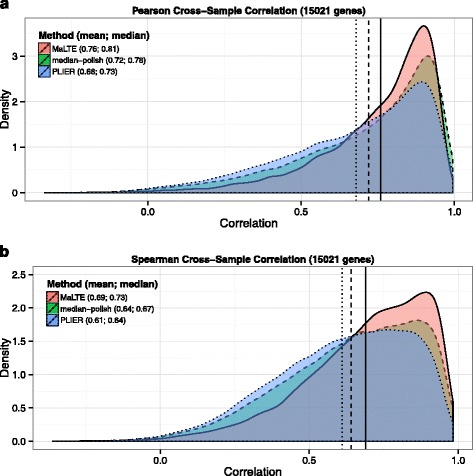
Cross-sample correlation with RNA-Seq. For each gene, the cross-sample correlation was determined between the gene expression values estimated from the microarrays using MaLTE, median-polish and PLIER. Density plots show the distribution across genes of (**a**) Pearson and (**b**) Spearman correlation coefficients. Mean and median values of the correlation coefficients are provided in parentheses next to the method name in the legend. Vertical lines show mean cross-sample correlation for MaLTE (solid), median-polish (dashed) and PLIER (dotted)

The correlation of microarray and RNA-Seq estimates of gene expression has been investigated previously in several studies [17, 32, 33]. Because not all genes vary substantially across samples, while within individual samples mRNA abundance ranges over several orders of magnitude [34], cross-sample correlations tend to be lower than within-sample correlations. MaLTE significantly outperformed median-polish and PLIER in cross-sample correlation (Fig. 3). For example, mean cross-sample Pearson correlation ($\bar {r}$), in test data was 0.76 for MaLTE compared to 0.72 for median-polish (*p*<1×10^−322^, from a Wilcoxon rank sum test of cross-sample correlations) and 0.68 for PLIER (Fig. 3a). Mean Spearman cross-sample correlations ($\bar {\rho }$) obtained from MaLTE were also much higher (0.69 compared to 0.64 for median-polish and 0.61 for PLIER; Fig. 3b).

### Restricting to well-estimated genes

**Fig. 4:**
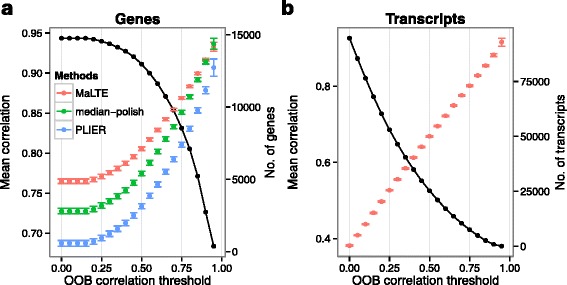
The effects of OOB filtering. Mean cross-sample Pearson correlation as a function of OOB correlation threshold for (**a**) genes and (**b**) transcripts. Error bars correspond to two standard errors. Note that transcript-level estimates are not provided by RMA and PLIER. The black line represents the number of genes/transcripts at each level

The performance of MaLTE is better for some genes. For example, low values of cross-sample correlation between MaLTE and RNA-Seq can be obtained for genes with low variation in expression across samples. Such genes will typically also show poor cross-sample correlation when their expression is estimated using median-polish and PLIER. However, MaLTE has the advantage that it provides an estimate of the accuracy with which the expression level of a given gene can be predicted. This is provided by the cross-validation carried out by Random Forest when the gene-specific regression model is learned from the training data [24]. Each regression tree in the forest is constructed from a subset of the samples. The expression level of the gene in a given sample can be estimated from the regression trees from which that sample was omitted. This is called the out-of-bag (OOB) estimate. For example, to estimate how well MaLTE will perform for a given gene as assessed by cross-sample correlation with RNA-Seq, we calculate the cross-sample correlation between the OOB estimates and the RNA-Seq data from the training samples. This provides an accurate estimate of the cross-sample correlation in test data (Supplementary Fig. S2). The OOB estimate can be used as a filter, so that MaLTE returns expression estimates only for genes with a desired property. By thresholding on the OOB cross-sample correlation, we found that very high values of cross-sample correlation can be achieved for a subset of genes (Fig. 4a). Because genes that pass the OOB cross-sample correlation threshold are likely to have high cross-sample variation, median-polish and PLIER also achieve higher cross-sample correlation for these genes. However, MaLTE maintains a performance advantage over the other methods with increasing threshold values (Fig. 4a).

### Inference of differential expression

For many gene expression studies a key objective is to identify the set of genes that are differentially expressed between groups of samples (e.g. disease versus non-disease or treatment versus control). To assess the performance of MaLTE in differential expression analysis we compared gene expression between two different GTEx tissues, heart muscle and skeletal muscle. RNA-Seq has been shown to have higher statistical power than microarrays for detecting differentially expressed genes [32]; therefore, we used similarity to the set of differentially expressed genes identified by RNA-Seq as the metric to evaluate the performance of MaLTE compared to median-polish and PLIER (Supplementary Fig. S3 and Fig. S4). To limit the influence of differences in platform-specific techniques for identifying differentially expressed genes (e.g. Cuffdiff for RNA-Seq, limma for microarrays) we used standard *t*-tests, and ranked the 21,367 common genes by the *q*-value. We measured the agreement between the ranked lists produced from the microarrays by alternative methods and from RNA-Seq using the concordance correlation coefficient (*ρ*_*c*_) and cumulative Jaccard index (see *Online Methods*; Supplementary Fig. S3a). MaLTE showed significantly higher concordance with RNA-Seq than median-polish or PLIER (*ρ*_*c*_=0.34, 0.16 and 0.12, respectively; Supplementary Fig. S3b), crucially demonstrating that MaLTE has performed better than these competing methods in identifying differentially expressed genes.

### Estimation of transcript isoform expression

A major advantage of RNA-Seq over microarrays is that RNA-seq can be used to discover and quantify the expression of novel transcript isoforms, resulting, for example, from alternative splicing. It is very difficult to obtain reliable estimates of expression at the level of transcript isoforms from gene expression microarrays, although some tentative methods have been proposed [35–37]. MaLTE extends naturally to the estimation of the abundance of specific isoforms by replacing gene expression as the fitted variable with transcript expression. Although it contains a lower density of probes than Affymetrix exon microarrays, the Affymetrix Human Gene 1.1 ST array contains multiple probe sets along human genes and can be used to measure exon inclusion [38]. Using multiple response regression we learned the relationship between the expression level of multiple transcript isoforms estimated from the RNA-Seq data and the fluorescence intensity of all probes mapping to the gene. We predicted the expression of 140,212 transcript isoforms, achieving mean cross-sample correlation with RNA-Seq estimates of 0.29. Again, the OOB expression estimates enabled us to identify smaller sets of transcript isoforms whose variation across samples can be predicted more accurately (Fig 4b). Using this approach, expression can be measured very accurately for about 25,000 transcript isoforms, which have a mean Pearson cross-sample correlation of over 0.65 (Fig. 4b and Supplementary Fig. S5).

### Application of MaLTE trained on GTEx data to independent microarray datasets

**Fig. 5:**
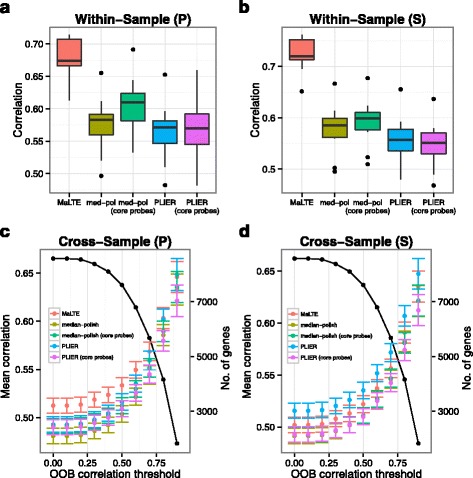
Application to archived data. MaLTE, trained using the GTEx data, was applied to predict gene expression from published microarray data based on brain samples for which RNA-Seq data was also available. Despite the fact that the two studies used different array platforms (Affymetrix Human Exon 1.0 ST arrays and Affymetrix Human Gene 1.1 ST arrays for the brain and GTEx studies, respectively), MaLTE predictions exceeded the within-sample correlations obtained using median-polish and PLIER. MaLTE predictions were based on probes shared between the two array platforms. Box plots of (**a**) Pearson and (**b**) Spearman within correlations are shown. (**c**) Pearson and (**d**) Spearman cross sample correlations with OOB filtering. The black line represents the number of genes/transcripts at each level

To determine whether MaLTE regression models, trained on a diverse panel of GTEx tissues, can be applied to estimate expression from microarrays generated independently we downloaded a dataset, consisting of Affymetrix Human Exon 1.0 ST microarrays and RNA-Seq data from a set of brain samples from a recent study [28]. The fact that these data were from a different array platform (an exon rather than gene array) posed a particular challenge, requiring that we restrict MaLTE to the subset of probes that are shared between the platforms (425,268 of 5,432,523 of the exon array probes are on the gene array). In spite of this, MaLTE again provided dramatic improvements in within-sample correlations compared to median-polish and PLIER and similar performance in cross-sample correlation (Figs. 5 and Supplementary Fig. S6). For this comparison, all of the methods used only the set of probes shared between the platforms because these are the only probes available to MaLTE. Without this restriction, the cross-sample correlations obtained using median-polish and PLIER applied to all core probe sets were, in fact, lower than for MaLTE. This is likely to be the result of noise resulting from lower quality probe sets that are not shared between the two platforms. Indeed the majority of exon array probes have been shown to contribute little to expression signals [38].

### Evaluation of MaLTE applied to a controlled tissue mixture dataset

Both of the evaluations of MaLTE discussed thus far involve comparison of expression estimates learned from microarrays with RNA-Seq estimates. This is the primary evaluation because the objective of MaLTE is to use arrays to approximate RNA-Seq (or any gold-standard measure that replaces it and for which a suitable training dataset is available). However, we also applied MaLTE to a tissue mixture dataset, provided by Affymetrix (http://www.affymetrix.com), for which only microarray data are available. The dataset consists of expression estimates from nine mixtures of commercially available heart and brain total RNA, with proportions 1:0 (pure heart), 0.95:0.05, 0.9:0.1, 0.5:0.5 (three biological replicates), 0.1:0.9, 0.05:0.95 and 0:1 (pure brain). At each mixture ratio three technical replicates were conducted, for a total of 33 arrays. Tissues were assayed using Affymetrix Human Gene 1.0 ST arrays. There were 9,455 genes that were called differentially expressed (FDR<0.05) between the pure heart and pure brain samples with each of the three methods. In the absence of biological noise and measurement error we expect to find a perfect linear correlation between the tissue proportion (i.e. brain or heart proportion) and the expression level for these genes. Because the biological noise is shared between the methods, the Pearson correlation coefficient gives an estimate of how consistent the gene expression measurements are across different sample mixtures. In this test, we would expect PLIER and RMA to outperform MaLTE because PLIER and RMA directly summarize the probe intensities, whereas MaLTE allows more complex relationships between expression levels of different probes and the gene expression estimate. The key advantage of MaLTE is that it provides an estimate of the absolute expression level, whereas, although the RMA and PLIER estimates are consistent across tissue mixtures, their relationship with actual gene expression level can be unclear. Nonetheless, the mean absolute value of the Pearson correlation coefficient from MaLTE was similar to that of RMA and PLIER (0.839 versus 0.874 and 0.896 for MaLTE, RMA and PLIER, respectively). Because MaLTE provides gene expression estimates on the absolute scale, characteristic of RNA-Seq it has significant advantages over the other methods in certain settings. For example, MaLTE outperformed the other methods when we applied CellMix [31], to estimate the tissue mixture proportions from the expression data, using gene expression deconvolution techniques [39]. The correlation between true and estimated proportions was high for all methods (Supplementary Fig. S7), but values estimated using MaLTE were closest to the true proportions (the slope of the regression line was 1.02 for MaLTE, compared to 0.86 and 0.96 for RMA and PLIER, respectively; Supplementary Fig. S7). Both Spearman and Pearson correlation coefficients between the true and estimated proportions were also highest for the estimates from MaLTE. In all cases filtering was applied (using OOB and removal of noisy genes; see *Online Methods*) at the training stage to determine which genes could be estimated accurately by MaLTE, but expression estimates for the same set of genes were provided to CellMix for each of the array estimation techniques.

### Running time

A parallel implementation of MaLTE is provided to facilitate application to large gene expression datasets. We timed MaLTE, RMA and PLIER on the task of estimating expression of 22,704 genes in 837 samples, using a double dual opteron compute node (quad core, 2.3GHz). RMA completed this task in 36 minutes, MaLTE (distributed over 12 cores) took 2 hours, 19 minutes and PLIER took 6.3 days.

## Discussion

Oligonucleotide expression microarrays frequently include multiple probes targeting genes or parts of genes. For these arrays, a key consideration is how to summarize fluorescence intensity signals from multiple probes in order to arrive at an estimate of the feature (e.g. gene or exon) of interest. A wide range of strategies to evaluate the performance of different microarray analysis tools and pipelines have been developed [3, 4]. We propose an alternative method to estimate gene and transcript expression from microarrays as well as a very different approach to the evaluation of performance. Given a gold standard measure of gene expression our goal is to obtain expression estimates from the microarray data that approximate it as closely as possible. The evaluation of performance then becomes a matter of determining how closely the expression estimates derived from the microarrays match the gold standard estimates. In principal any high-throughput measure of gene expression can play the role of gold standard, provided a sufficiently large and diverse set of training samples with both microarray and gold standard expression data is available. Here we use RNA-Seq because it has been shown to have several important advantages over microarrays [10, 40]. Our goal is to estimate gene expression from microarrays in a way that attains some of these advantages. To achieve this, we learned the relationship between RNA-Seq transcript expression levels and microarray probe intensities from a subset of the GTEx [14] samples, evaluating performance on the remainder, as well as on data from a second study [28] that also generated RNA-Seq and microarrays from the same set of samples.

Compared to two widely-used methods to estimate expression levels from microarrays, MaLTE obtained much greater within-sample correlation with RNA-Seq estimates on both the test GTEx data [14] and on the brain dataset [28] and comparable (for the brain dataset) or significantly better (for the GTEx test data) cross-sample correlation. The performance on the brain dataset was achieved in spite of the use of very different array platforms for the training and test samples in this case that shared only a small proportion of probes (an exon microarray for the brain dataset and a gene microarray for GTEx). In addition to high within-sample correlation coefficients, the slopes of the regression lines of MaLTE against RNA-Seq for each sample were close to one (Supplementary Fig. S6). Taken together, this indicates that MaLTE provides an estimate of the RNA-Seq data on the same scale as RNA-Seq. MaLTE can be applied in the context of large studies where RNA-Seq and arrays are applied to a subset of the samples and arrays only to the remainder. The performance on the Mazin dataset suggests that MaLTE, trained on the GTEx data, can also be applied to archived samples, despite batch effects and, in this case, differences in array platforms. Further improvements in performance are likely to be possible if batch effects are modeled appropriate and methods exist for this purpose.

Tissue-specific alternative splicing can complicate the relationship between the signal from a collection of probes and gene expression level because different transcript isoforms may dominate in different tissues. We have found (data not shown) that the performance of MaLTE can be further improved by first carrying out principal component analysis on the probe intensity matrix and including a subset of the principal components as features that MaLTE uses to predict gene expression. In this case the feature set for a given gene includes shared (experiment-wide) principal components in addition to the gene-specific probe intensities.

## Conclusion

Our results show that MaLTE, trained on the GTEx dataset, can be applied to estimate gene expression accurately from microarray data generated in other studies. There are currently over 24,000 expression microarray datasets in the GEO database [41] including more than 9,000 from humans. Affymetrix GeneChip gene and exon array platforms together account for over 1,100 expression array experiments, involving over 19,000 samples. MaLTE offers an alternative approach to the analysis of these data, which will allow the estimated gene and transcript expression levels to become more comparable with expression estimates from RNA-Seq. Archived microarray datasets represent a rich resource of data and, by learning the relationship between probe intensities and RNA-Seq expression estimates, MaLTE offers the possibility of joint analysis of data generated using RNA-Seq and microarrays. In general, training datasets that have been assayed using different platforms represent a Rosetta Stone for gene expression measurement, allowing measurements from one platform to be translated to another. Due to the study size and the breadth of sample types, GTEx serves this role for Affymetrix gene oligonucleotide arrays and RNA-Seq.

## Additional file

Additional file 1
**Additional information containing online methods is provided as a PDF file.** **Figure S1.** The effect of training size on predictions. A test set of 100 samples was used for all training sample sizes. **Figure S2.** Out-of-bag (OOB) filtering. Scatter plot of cross-sample correlation coefficients from on OOB gene expression estimates and estimates obtained on test data for 1,000 randomly selected genes. **Figure S3.** Comparison of differential expression results. Comparison of the performance of MaLTE and median-polish on the problem of detecting differential gene expression between five heart-muscle and five skeletal-muscle samples in the GTEx dataset. Each method results in a list of genes, ranked by the *q*-value from the comparison of the gene expression level in the two groups of samples. We used the (**a**) cumulative Jaccard index and (**b**) concordance correlation to compare the similarity as a function of rank and the overall similarity, respectively, between lists of genes ranked by MaLTE/median-polish and by RNA-seq. The set of genes assessed were defined by RNA-Seq: genes with RPKM of above one in both tissues. Bootstrap re-sampling (100 pseudo-replicates) was used to assess the effect of sampling error for both cumulative Jaccard index and concordance correlations. Results of bootstrapping are shown as faint lines around the observed Jaccard index and yield the density plots shown in (**b**). Log of fold change estimates for (**c**) 120 differentially expressed genes common to MaLTE and median-polish and (**d**) MaLTE and PLIER for six common genes. **Figure S4.** Comparison of 10-sample DGE to 44-sample DGE. (**a**) Self-comparisons (e.g. MaLTE 10-sample DGE to MaLTE 44-sample DGE, with five and 22 samples in each tissue, respectively). Forty-four-sample DGE results are treated as putative true differential expressions at the indicated (top right or each plot) false discover rates (FDRs). FDRs were computed using the *q*-value method (Storey and Tibshirani 2003). Comparisons are made using the Jaccard index. (**b**) Comparison of each method to 44-sample DGE using RNA-Seq. **Figure S5.** Transcript isoform expression estimates. Densities of (**a**) Pearson and (**b**) Spearman cross-sample correlations for transcript isoform expression estimates obtained using MaLTE. Filtered data corresponds to transcript isoforms with *r*_OOB_>0. **Figure S6.** Box plots of slopes *β* computed by linearly regressing RNA-Seq against array method. Dotted red line indicates unit gradient. Each box plot represents 12 slopes for brain samples. RNA-Seq expression is restricted to RPKM between one and 1000 because of high uncertainty at low values and few genes at high values representing 78% of genes quantified. Using all genes results in the same medians but wider variance in slopes due to outlying genes. **Figure S7.** Estimation of tissue mixture proportions. (**a**) MaLTE, (**b**) median-polish (RMA), and (**c**) PLIER. Each plot shows comparisons of true and estimated proportions with key statistics indicated in the legends.

## References

[1] Johnson JM, Castle J, Garrett-Engele P, Kan Z, Loerch PM, Armour CD, Santos R, Schadt EE, Stoughton R, Shoemaker DD (2003). Genome-wide survey of human alternative pre-mRNA splicing with exon junction microarrays. Science.

[2] Irizarry RA, Bolstad BM, Collin F, Cope LM, Hobbs B, Speed TP (2003). Summaries of Affymetrix GeneChip probe level data. Nucleic Acids Research.

[3] Irizarry RA, Wu Z, Jaffee HA (2006). Comparison of Affymetrix GeneChip expression measures. Bioinformatics.

[4] Choe SE, Boutros M, Michelson AM, Church GM, Halfon MS (2005). Preferred analysis methods for Affymetrix GeneChips revealed by a wholly defined control dataset. Genome Biology.

[5] Miller JA, Menon V, Goldy J, Kaykas A, Lee C-K, Smith KA, Shen EH, Phillips JW, Lein ES, Hawrylycz MJ (2014). Improving reliability and absolute quantification of human brain microarray data by filtering and scaling probes using RNA-Seq. BMC genomics.

[6] Fu X, Fu N, Guo S, Yan Z, Xu Y, Hu H, Menzel C, Chen W, Li Y, Zeng R (2009). Estimating accuracy of RNA-Seq and microarrays with proteomics. BMC Genomics.

[7] Mutch DM, Berger A, Mansourian R, Rytz A, Roberts M-A (2002). The limit fold change model: a practical approach for selecting differentially expressed genes from microarray data. BMC Bioinformatics.

[8] Irizarry RA, Warren D, Spencer F, Kim IF, Biswal S, Frank BC, Gabrielson E, Garcia JG, Geoghegan J, Germino G (2005). Multiple-laboratory comparison of microarray platforms. Nature Methods.

[9] Seita J, Sahoo D, Rossi DJ, Bhattacharya D, Serwold T, Inlay MA, Ehrlich LI, Fathman JW, Dill DL, Weissman IL (2012). Gene expression commons: an open platform for absolute gene expression profiling. PLoS ONE.

[10] Wang Z, Gerstein M, Snyder M (2009). RNA-Seq: a revolutionary tool for transcriptomics. Nature Reviews Genetics.

[11] Birney E, Stamatoyannopoulos JA, Dutta A, Guigó R, Gingeras TR, Margulies EH, Weng Z, Snyder M, Dermitzakis ET, Thurman RE (2007). Identification and analysis of functional elements in 1% of the human genome by the ENCODE pilot project. Nature.

[12] Meyer S, Fuchs TJ, Bosserhoff AK, Hofstädter F, Pauer A, Roth V, Buhmann JM, Moll I, Anagnostou N, Brandner JM (2012). A seven-marker signature and clinical outcome in malignant melanoma: a large-scale tissue-microarray study with two independent patient cohorts. PLoS ONE.

[13] Clarke C, Doolan P, Barron N, Meleady P, O’Sullivan F, Gammell P, Melville M, Leonard M, Clynes M (2011). Large scale microarray profiling and coexpression network analysis of CHO cells identifies transcriptional modules associated with growth and productivity. Journal of Biotechnology.

[14] Lonsdale J, Thomas J, Salvatore M, Phillips R, Lo E, Shad S, Hasz R, Walters G, Garcia F, Young N (2013). The Genotype-Tissue Expression (GTEx) project. Nature Genetics.

[15] Affymetrix. Technical note: guide to probe logarithmic intensity error (PLIER) estimation. Technical report, Affymetrix Inc. 2005. http://www.affymetrix.com/support/technical/technotes/plier_technote.pdf Accessed 2013-04-22.

[16] Flicek P, Amode MR, Barrell D, Beal K, Brent S, Carvalho-Silva D, Clapham P, Coates G, Fairley S, Fitzgerald S (2012). Ensembl 2012. Nucleic Acids Research.

[17] Pickrell JK, Marioni JC, Pai AA, Degner JF, Engelhardt BE, Nkadori E, Veyrieras J-B, Stephens M, Gilad Y, Pritchard JK (2010). Understanding mechanisms underlying human gene expression variation with RNA sequencing. Nature.

[18] Montgomery SB, Sammeth M, Gutierrez-Arcelus M, Lach RP, Ingle C, Nisbett J, Guigo R, Dermitzakis ET (2010). Transcriptome genetics using second generation sequencing in a Caucasian population. Nature.

[19] Huang RS, Duan S, Bleibel WK, Kistner EO, Zhang W, Clark TA, Chen TX, Schweitzer AC, Blume JE, Cox NJ (2007). A genome-wide approach to identify genetic variants that contribute to etoposide-induced cytotoxicity. Proceedings of the National Academy of Sciences.

[20] Gibbs RA, Belmont JW, Hardenbol P, Willis TD, Yu F, Yang H, Ch’ang L-Y, Huang W, Liu B, Shen Y (2003). The international HapMap project. Nature.

[21] Edgar R, Domrachev M, Lash AE (2002). Gene Expression Omnibus: NCBI gene expression and hybridization array data repository. Nucleic Acids Research.

[22] Breiman L (1993). Classification and Regression Trees.

[23] Friedamn JH (1991). Multivariate adaptive regression splines. The Annals of Statistics.

[24] Breiman L (2001). Random forests. Machine Learning.

[25] Hothorn T, Hornik K, Zeileis A (2006). Unbiased recursive partitioning: A conditional inference framework. Journal of Computational and Graphical statistics.

[26] Meinshausen N (2006). Quantile regression forests. The Journal of Machine Learning Research.

[27] Team RC. R: A language and environment for statistical computing. ISBN 3-900051-07-0 R Foundation for Statistical Computing Vienna, Austria, 2013. (2005) http://www.r-project.org.

[28] Mazin P, Xiong J, Liu X, Yan Z, Zhang X, Li M, He L, Somel M, Yuan Y, Phoebe CY (2013). Widespread splicing changes in human brain development and aging. Molecular systems biology.

[29] Affymetrix. Exon Array Whitepaper Collection,“Exon Probeset Annotations and Transcript Cluster Groupings,” rev. Sep. 27, 2005, ver. 1.0. Technical report, Affymetrix Inc. (2005). http://www.affymetrix.com/support/technical/whitepapers/exon_probeset_trans_clust_whitepaper.pdf.

[30] Yates T, Okoniewski MJ, Miller CJ (2008). X:Map: annotation and visualization of genome structure for Affymetrix exon array analysis. Nucleic Acids Research.

[31] Gaujoux R, Seoighe C. CellMix: A Comprehensive Toolbox for Gene Expression Deconvolution. Bioinformatics. 2013. doi:10.1093/bioinformatics/btt351. 10.1093/bioinformatics/btt351 23825367

[32] Marioni JC, Mason CE, Mane SM, Stephens M, Gilad Y (2008). RNA-Seq: an assessment of technical reproducibility and comparison with gene expression arrays. Genome Research.

[33] Mortazavi A, Williams BA, McCue K, Schaeffer L, Wold B (2008). Mapping and quantifying mammalian transcriptomes by RNA-Seq. Nature Methods.

[34] Schwanhäusser B, Busse D, Li N, Dittmar G, Schuchhardt J, Wolf J, Chen W, Selbach M (2011). Global quantification of mammalian gene expression control. Nature.

[35] Kwan T, Benovoy D, Dias C, Gurd S, Provencher C, Beaulieu P, Hudson TJ, Sladek R, Majewski J (2008). Genome-wide analysis of transcript isoform variation in humans. Nature Genetics.

[36] Turro E, Lewin A, Rose A, Dallman MJ, Richardson S (2010). MMBGX: a method for estimating expression at the isoform level and detecting differential splicing using whole-transcript Affymetrix arrays. Nucleic Acids Research.

[37] Anton MA, Gorostiaga D, Guruceaga E, Segura V, Carmona-Saez P, Pascual-Montano A, Pio R, Montuenga LM, Rubio A (2008). SPACE: an algorithm to predict and quantify alternatively spliced isoforms using microarrays. Genome Biology.

[38] Robinson MD, Speed TP (2007). A comparison of Affymetrix gene expression arrays. BMC Bioinformatics.

[39] Shen-Orr SS, Tibshirani R, Khatri P, Bodian DL, Staedtler F, Perry NM, Hastie T, Sarwal MM, Davis MM, Butte AJ (2010). Cell type–specific gene expression differences in complex tissues. Nature methods.

[40] Ozsolak F, Milos PM (2010). RNA sequencing: advances, challenges and opportunities. Nature Reviews Genetics.

[41] Barrett T, Troup DB, Wilhite SE, Ledoux P, Rudnev D, Evangelista C, Kim IF, Soboleva A, Tomashevsky M, Edgar R (2007). NCBI GEO: mining tens of millions of expression profiles-database and tools update. Nucleic Acids Research.

