# Supplementary figures and images for: Seq-ing improved gene expression estimates from microarrays using machine learning
Source: BMC Bioinformatics. 2015 Sep 4;16:286. doi: 10.1186/s12859-015-0712-z (PMC4559919; doi:10.1186/s12859-015-0712-z)

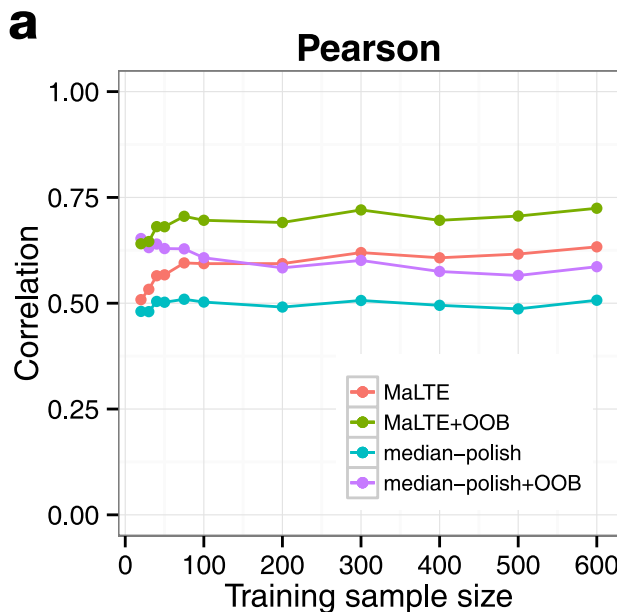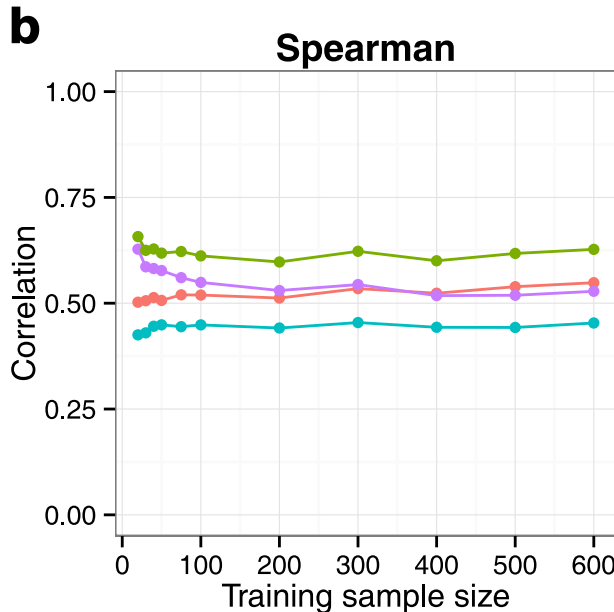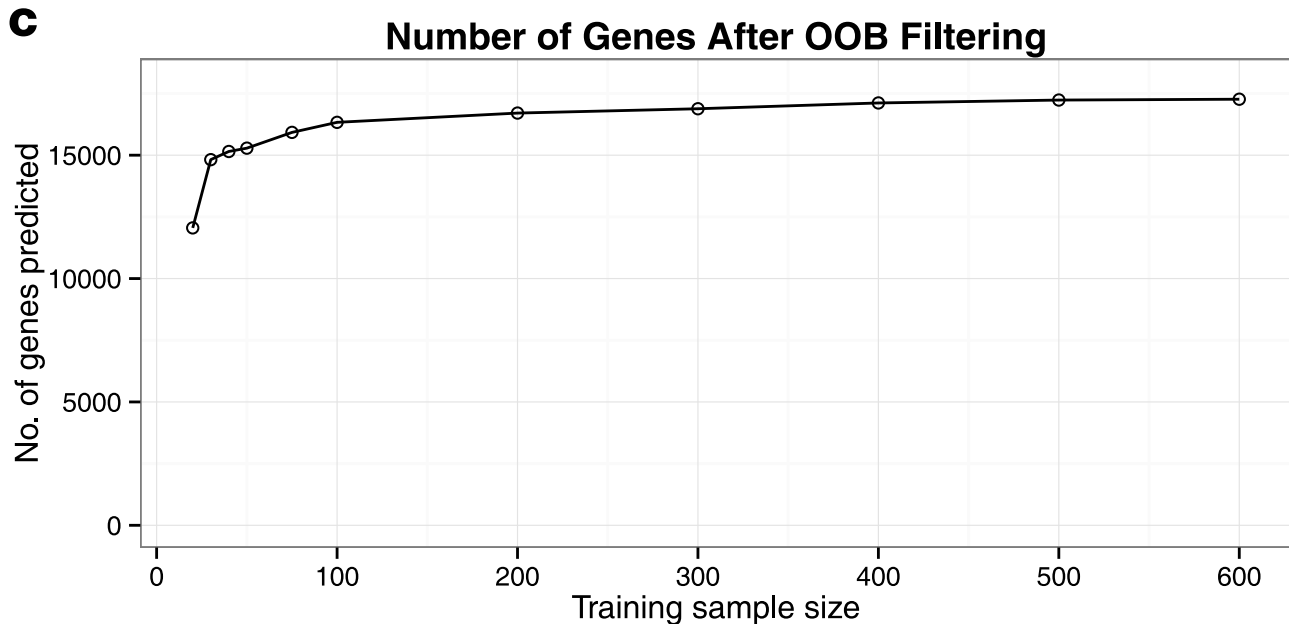

Supplement: Additional file 1 — Additional information containing online methods is provided as a PDF file. Figure S1. The effect of training size on predictions. A test set of 100 samples was used for all training sample sizes. Figure S2. Out-of-bag (OOB) filtering. Scatter plot of cross-sample correlation coefficients from on OOB gene expression estimates and estimates obtained on test data for 1,000 randomly selected genes. Figure S3. Comparison of differential expression results. Comparison of the performance of MaLTE and median-polish on the problem of detecting differential gene expression between five heart-muscle and five skeletal-muscle samples in the GTEx dataset. Each method results in a list of genes, ranked by the q-value from the comparison of the gene expression level in the two groups of samples. We used the (a) cumulative Jaccard index and (b) concordance correlation to compare the similarity as a function of rank and the overall similarity, respectively, between lists of genes ranked by MaLTE/median-polish and by RNA-seq. The set of genes assessed were defined by RNA-Seq: genes with RPKM of above one in both tissues. Bootstrap re-sampling (100 pseudo-replicates) was used to assess the effect of sampling error for both cumulative Jaccard index and concordance correlations. Results of bootstrapping are shown as faint lines around the observed Jaccard index and yield the density plots shown in (b). Log of fold change estimates for (c) 120 differentially expressed genes common to MaLTE and median-polish and (d) MaLTE and PLIER for six common genes. Figure S4. Comparison of 10-sample DGE to 44-sample DGE. (a) Self-comparisons (e.g. MaLTE 10-sample DGE to MaLTE 44-sample DGE, with five and 22 samples in each tissue, respectively). Forty-four-sample DGE results are treated as putative true differential expressions at the indicated (top right or each plot) false discover rates (FDRs). FDRs were computed using the q-value method (Storey and Tibshirani 2003). Comparisons are made using the [file 12859_2015_712_MOESM1_ESM.pdf › MOESM1/figureS1_GTEx_QRF.pdf]

# OOB Filtering

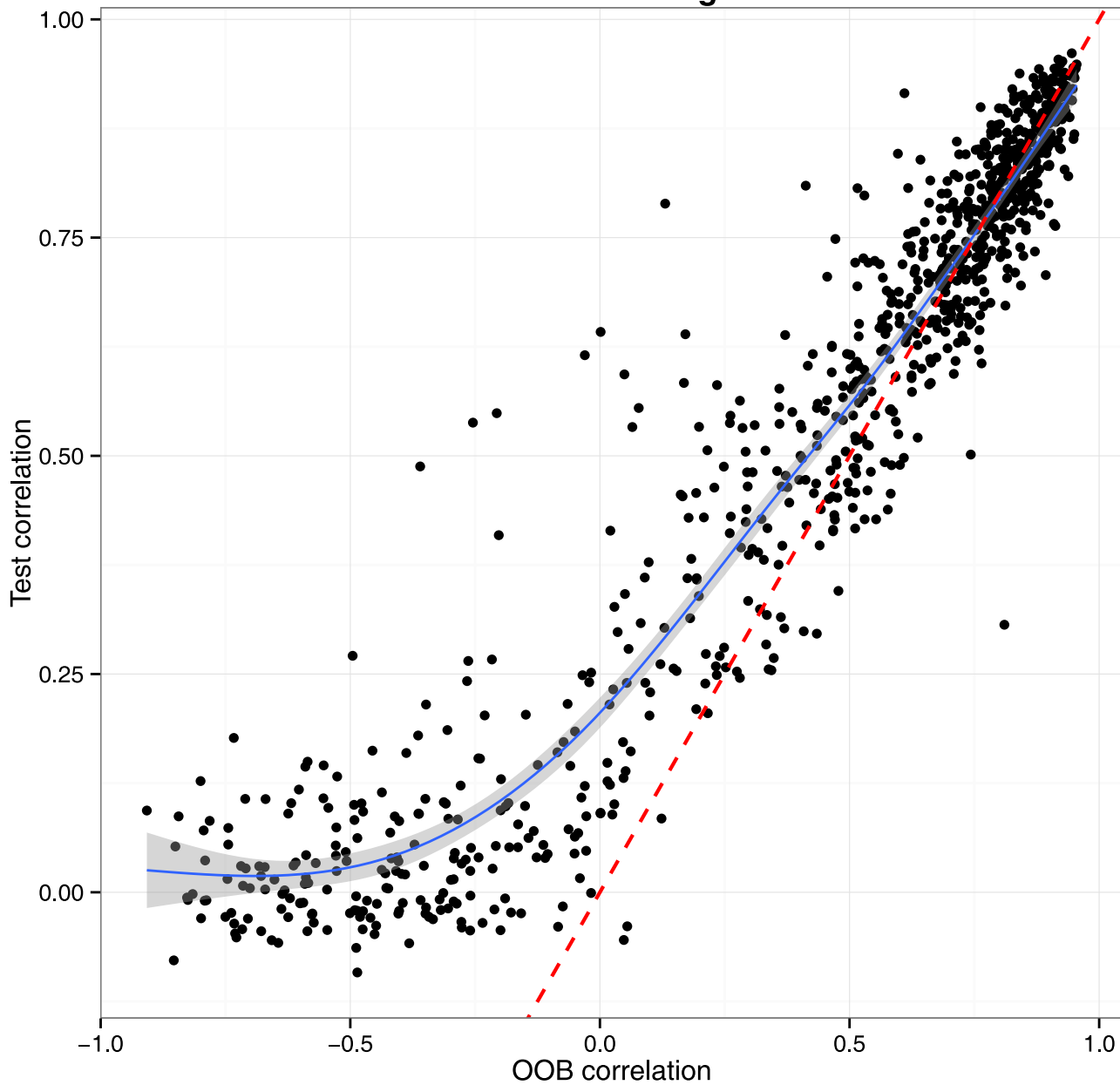

Supplement: Additional file 1 — Additional information containing online methods is provided as a PDF file. Figure S1. The effect of training size on predictions. A test set of 100 samples was used for all training sample sizes. Figure S2. Out-of-bag (OOB) filtering. Scatter plot of cross-sample correlation coefficients from on OOB gene expression estimates and estimates obtained on test data for 1,000 randomly selected genes. Figure S3. Comparison of differential expression results. Comparison of the performance of MaLTE and median-polish on the problem of detecting differential gene expression between five heart-muscle and five skeletal-muscle samples in the GTEx dataset. Each method results in a list of genes, ranked by the q-value from the comparison of the gene expression level in the two groups of samples. We used the (a) cumulative Jaccard index and (b) concordance correlation to compare the similarity as a function of rank and the overall similarity, respectively, between lists of genes ranked by MaLTE/median-polish and by RNA-seq. The set of genes assessed were defined by RNA-Seq: genes with RPKM of above one in both tissues. Bootstrap re-sampling (100 pseudo-replicates) was used to assess the effect of sampling error for both cumulative Jaccard index and concordance correlations. Results of bootstrapping are shown as faint lines around the observed Jaccard index and yield the density plots shown in (b). Log of fold change estimates for (c) 120 differentially expressed genes common to MaLTE and median-polish and (d) MaLTE and PLIER for six common genes. Figure S4. Comparison of 10-sample DGE to 44-sample DGE. (a) Self-comparisons (e.g. MaLTE 10-sample DGE to MaLTE 44-sample DGE, with five and 22 samples in each tissue, respectively). Forty-four-sample DGE results are treated as putative true differential expressions at the indicated (top right or each plot) false discover rates (FDRs). FDRs were computed using the q-value method (Storey and Tibshirani 2003). Comparisons are made using the [file 12859_2015_712_MOESM1_ESM.pdf › MOESM1/figureS2_GTEx_QRF.pdf]

**a**

### Similarity to RNA-Seq

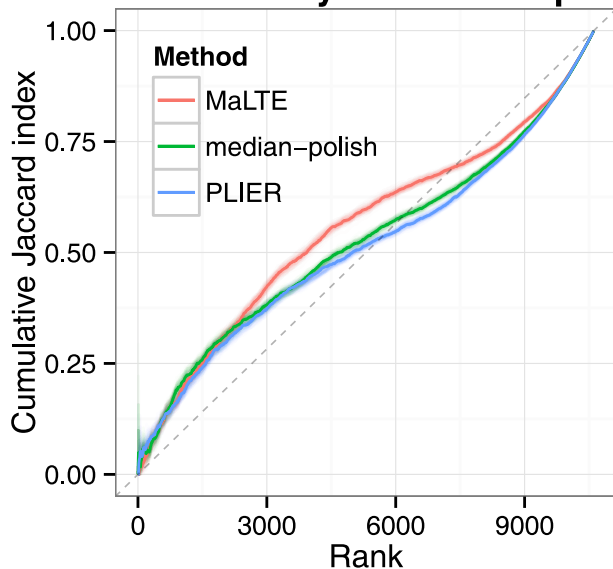**b**

### Concordance

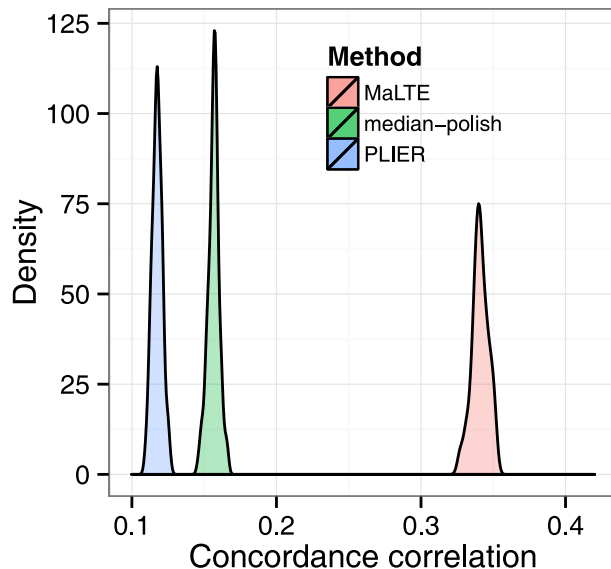**c**

### Log of Fold Change

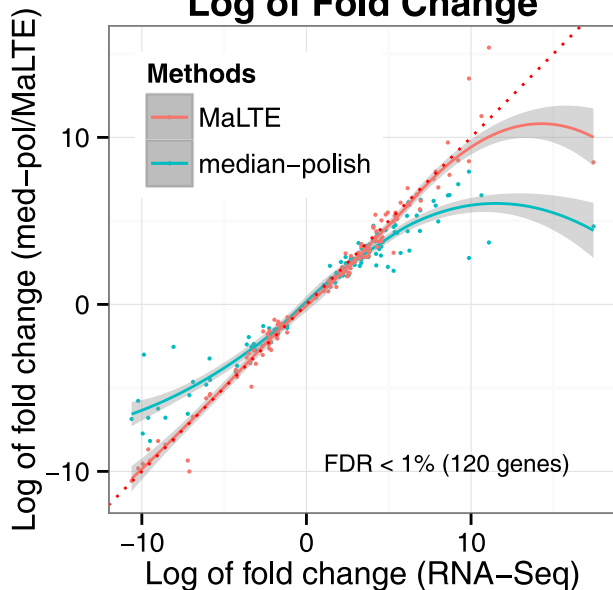**d**

### Log of Fold Change

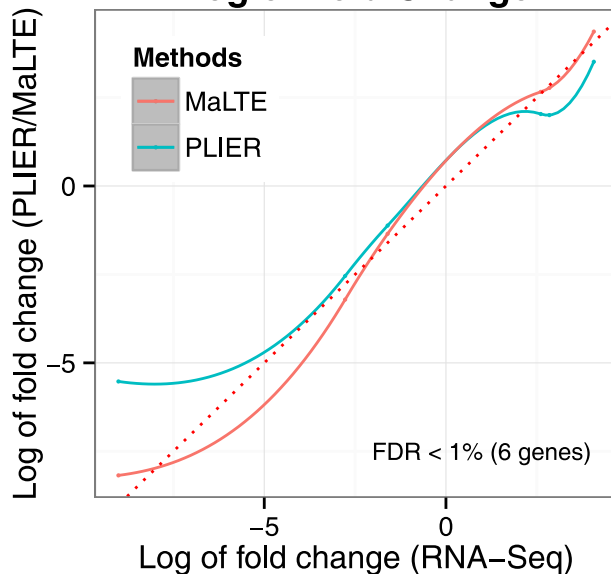

Supplement: Additional file 1 — Additional information containing online methods is provided as a PDF file. Figure S1. The effect of training size on predictions. A test set of 100 samples was used for all training sample sizes. Figure S2. Out-of-bag (OOB) filtering. Scatter plot of cross-sample correlation coefficients from on OOB gene expression estimates and estimates obtained on test data for 1,000 randomly selected genes. Figure S3. Comparison of differential expression results. Comparison of the performance of MaLTE and median-polish on the problem of detecting differential gene expression between five heart-muscle and five skeletal-muscle samples in the GTEx dataset. Each method results in a list of genes, ranked by the q-value from the comparison of the gene expression level in the two groups of samples. We used the (a) cumulative Jaccard index and (b) concordance correlation to compare the similarity as a function of rank and the overall similarity, respectively, between lists of genes ranked by MaLTE/median-polish and by RNA-seq. The set of genes assessed were defined by RNA-Seq: genes with RPKM of above one in both tissues. Bootstrap re-sampling (100 pseudo-replicates) was used to assess the effect of sampling error for both cumulative Jaccard index and concordance correlations. Results of bootstrapping are shown as faint lines around the observed Jaccard index and yield the density plots shown in (b). Log of fold change estimates for (c) 120 differentially expressed genes common to MaLTE and median-polish and (d) MaLTE and PLIER for six common genes. Figure S4. Comparison of 10-sample DGE to 44-sample DGE. (a) Self-comparisons (e.g. MaLTE 10-sample DGE to MaLTE 44-sample DGE, with five and 22 samples in each tissue, respectively). Forty-four-sample DGE results are treated as putative true differential expressions at the indicated (top right or each plot) false discover rates (FDRs). FDRs were computed using the q-value method (Storey and Tibshirani 2003). Comparisons are made using the [file 12859_2015_712_MOESM1_ESM.pdf › MOESM1/figureS3_GTEx_QRF.pdf]

**a****Self comparison**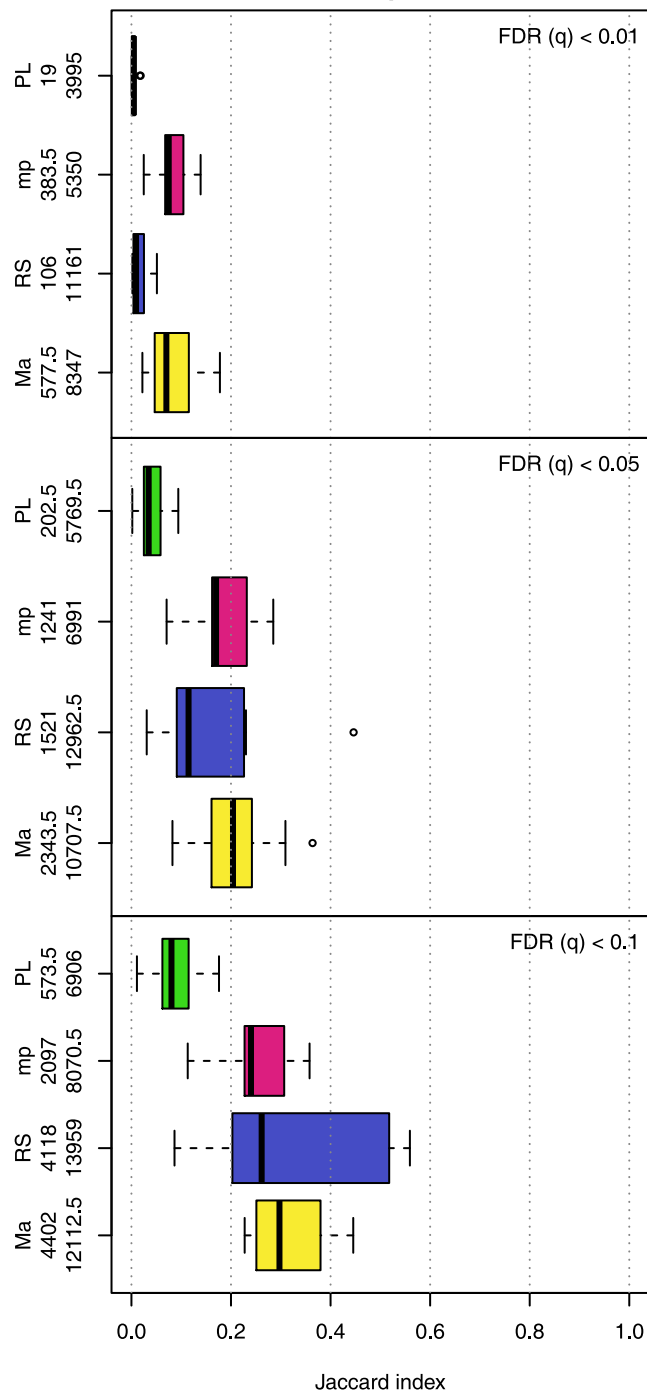**b****Comparison to RNA-Seq with 44 samples**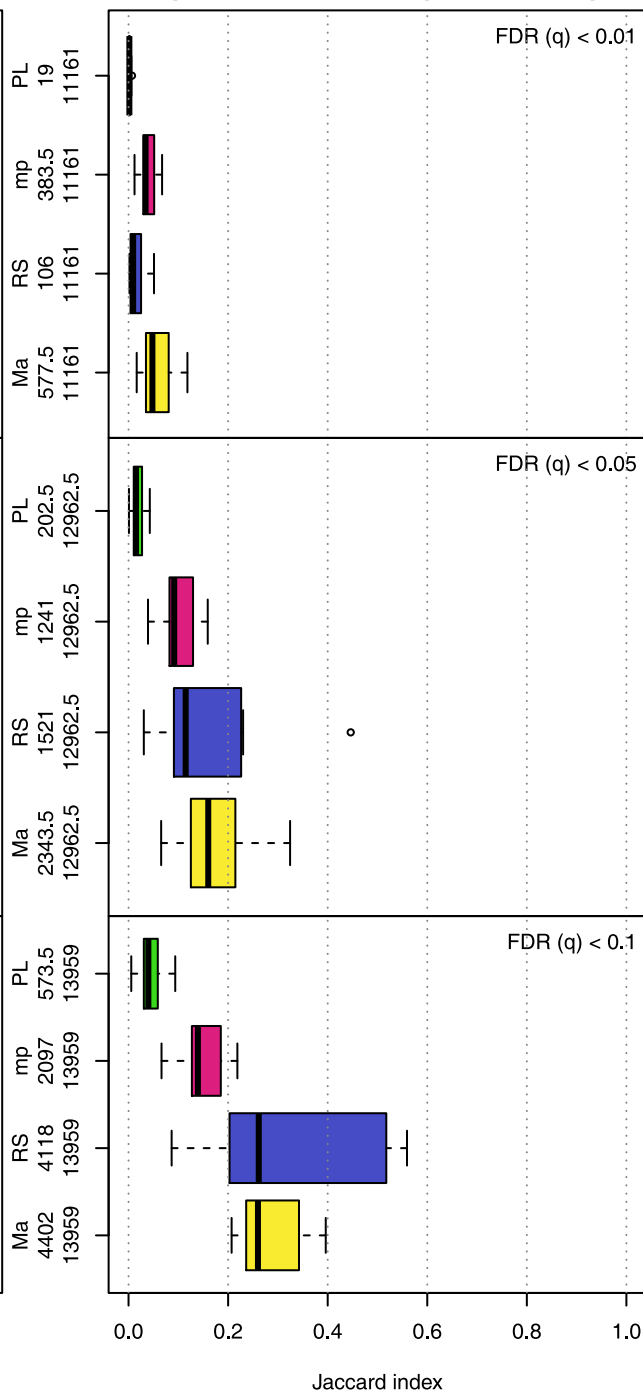

Supplement: Additional file 1 — Additional information containing online methods is provided as a PDF file. Figure S1. The effect of training size on predictions. A test set of 100 samples was used for all training sample sizes. Figure S2. Out-of-bag (OOB) filtering. Scatter plot of cross-sample correlation coefficients from on OOB gene expression estimates and estimates obtained on test data for 1,000 randomly selected genes. Figure S3. Comparison of differential expression results. Comparison of the performance of MaLTE and median-polish on the problem of detecting differential gene expression between five heart-muscle and five skeletal-muscle samples in the GTEx dataset. Each method results in a list of genes, ranked by the q-value from the comparison of the gene expression level in the two groups of samples. We used the (a) cumulative Jaccard index and (b) concordance correlation to compare the similarity as a function of rank and the overall similarity, respectively, between lists of genes ranked by MaLTE/median-polish and by RNA-seq. The set of genes assessed were defined by RNA-Seq: genes with RPKM of above one in both tissues. Bootstrap re-sampling (100 pseudo-replicates) was used to assess the effect of sampling error for both cumulative Jaccard index and concordance correlations. Results of bootstrapping are shown as faint lines around the observed Jaccard index and yield the density plots shown in (b). Log of fold change estimates for (c) 120 differentially expressed genes common to MaLTE and median-polish and (d) MaLTE and PLIER for six common genes. Figure S4. Comparison of 10-sample DGE to 44-sample DGE. (a) Self-comparisons (e.g. MaLTE 10-sample DGE to MaLTE 44-sample DGE, with five and 22 samples in each tissue, respectively). Forty-four-sample DGE results are treated as putative true differential expressions at the indicated (top right or each plot) false discover rates (FDRs). FDRs were computed using the q-value method (Storey and Tibshirani 2003). Comparisons are made using the [file 12859_2015_712_MOESM1_ESM.pdf › MOESM1/figureS4_GTEx_QRF.pdf]

**a****Pearson cross-sample correlations**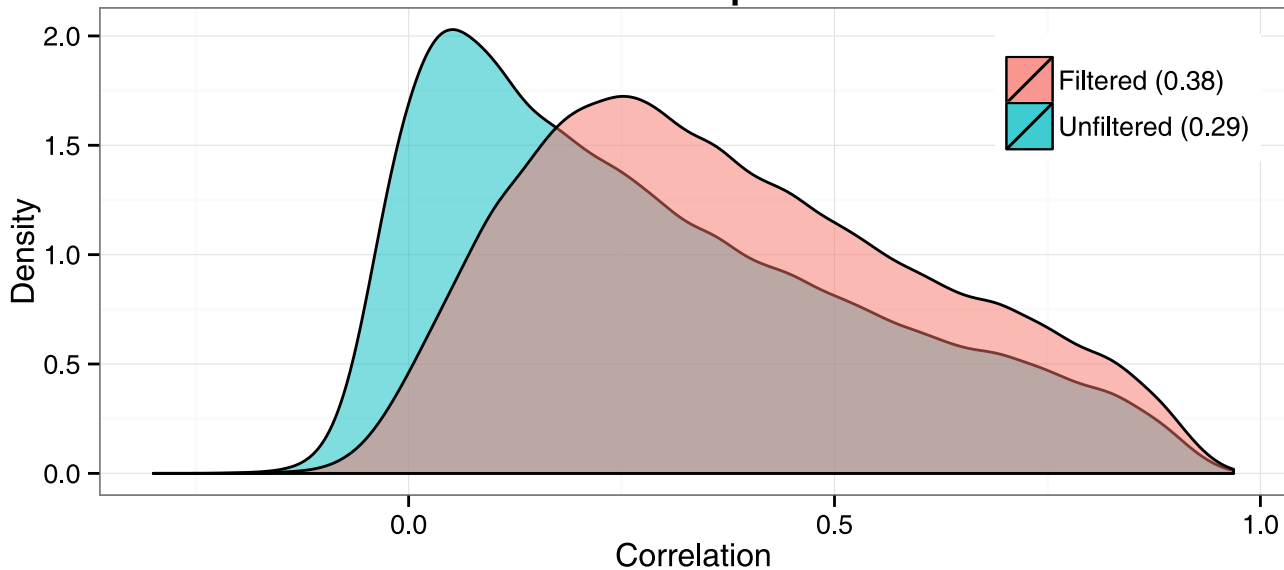**b****Spearman cross-sample correlations**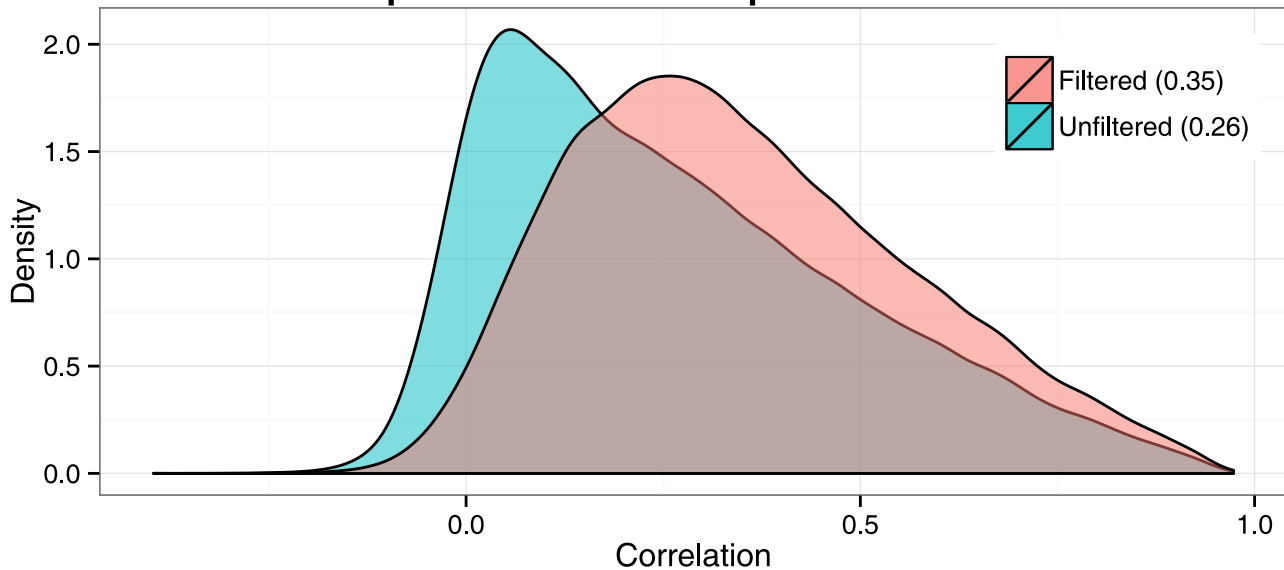

Supplement: Additional file 1 — Additional information containing online methods is provided as a PDF file. Figure S1. The effect of training size on predictions. A test set of 100 samples was used for all training sample sizes. Figure S2. Out-of-bag (OOB) filtering. Scatter plot of cross-sample correlation coefficients from on OOB gene expression estimates and estimates obtained on test data for 1,000 randomly selected genes. Figure S3. Comparison of differential expression results. Comparison of the performance of MaLTE and median-polish on the problem of detecting differential gene expression between five heart-muscle and five skeletal-muscle samples in the GTEx dataset. Each method results in a list of genes, ranked by the q-value from the comparison of the gene expression level in the two groups of samples. We used the (a) cumulative Jaccard index and (b) concordance correlation to compare the similarity as a function of rank and the overall similarity, respectively, between lists of genes ranked by MaLTE/median-polish and by RNA-seq. The set of genes assessed were defined by RNA-Seq: genes with RPKM of above one in both tissues. Bootstrap re-sampling (100 pseudo-replicates) was used to assess the effect of sampling error for both cumulative Jaccard index and concordance correlations. Results of bootstrapping are shown as faint lines around the observed Jaccard index and yield the density plots shown in (b). Log of fold change estimates for (c) 120 differentially expressed genes common to MaLTE and median-polish and (d) MaLTE and PLIER for six common genes. Figure S4. Comparison of 10-sample DGE to 44-sample DGE. (a) Self-comparisons (e.g. MaLTE 10-sample DGE to MaLTE 44-sample DGE, with five and 22 samples in each tissue, respectively). Forty-four-sample DGE results are treated as putative true differential expressions at the indicated (top right or each plot) false discover rates (FDRs). FDRs were computed using the q-value method (Storey and Tibshirani 2003). Comparisons are made using the [file 12859_2015_712_MOESM1_ESM.pdf › MOESM1/figureS5_GTEx_QRF.pdf]

$\beta$

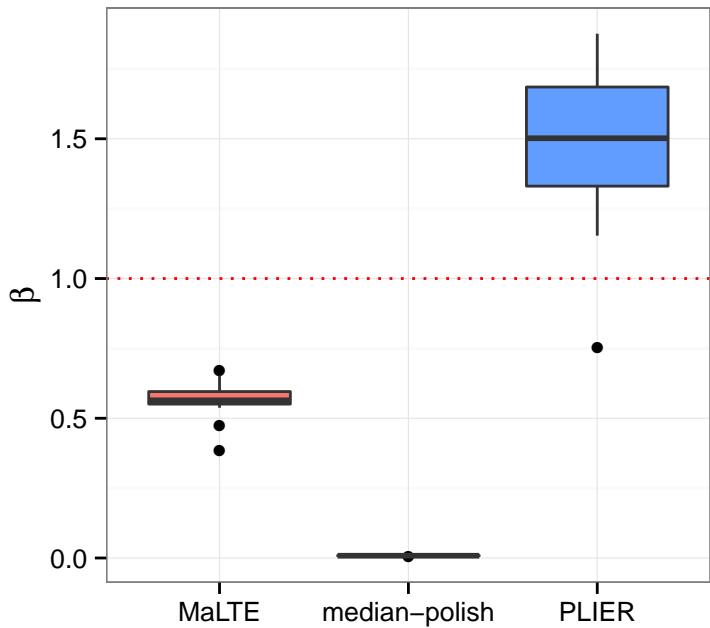

Supplement: Additional file 1 — Additional information containing online methods is provided as a PDF file. Figure S1. The effect of training size on predictions. A test set of 100 samples was used for all training sample sizes. Figure S2. Out-of-bag (OOB) filtering. Scatter plot of cross-sample correlation coefficients from on OOB gene expression estimates and estimates obtained on test data for 1,000 randomly selected genes. Figure S3. Comparison of differential expression results. Comparison of the performance of MaLTE and median-polish on the problem of detecting differential gene expression between five heart-muscle and five skeletal-muscle samples in the GTEx dataset. Each method results in a list of genes, ranked by the q-value from the comparison of the gene expression level in the two groups of samples. We used the (a) cumulative Jaccard index and (b) concordance correlation to compare the similarity as a function of rank and the overall similarity, respectively, between lists of genes ranked by MaLTE/median-polish and by RNA-seq. The set of genes assessed were defined by RNA-Seq: genes with RPKM of above one in both tissues. Bootstrap re-sampling (100 pseudo-replicates) was used to assess the effect of sampling error for both cumulative Jaccard index and concordance correlations. Results of bootstrapping are shown as faint lines around the observed Jaccard index and yield the density plots shown in (b). Log of fold change estimates for (c) 120 differentially expressed genes common to MaLTE and median-polish and (d) MaLTE and PLIER for six common genes. Figure S4. Comparison of 10-sample DGE to 44-sample DGE. (a) Self-comparisons (e.g. MaLTE 10-sample DGE to MaLTE 44-sample DGE, with five and 22 samples in each tissue, respectively). Forty-four-sample DGE results are treated as putative true differential expressions at the indicated (top right or each plot) false discover rates (FDRs). FDRs were computed using the q-value method (Storey and Tibshirani 2003). Comparisons are made using the [file 12859_2015_712_MOESM1_ESM.pdf › MOESM1/figureS6_GTEx_QRF.pdf]
